# Diagnostic and therapeutic features associated with modification of quality-of-life's outcomes between one and six months after major surgery for head and neck cancer^☆^

**DOI:** 10.1016/j.bjorl.2015.10.013

**Published:** 2015-12-18

**Authors:** Margherita Gobbo, Federica Bullo, Giuseppe Perinetti, Annalisa Gatto, Giulia Ottaviani, Matteo Biasotto, Giancarlo Tirelli

**Affiliations:** a Dental Science Department, Division of Oral Medicine and Pathology, Trieste, Italy; b University of Trieste, Hospital of Cattinara, ENT Clinic, Head and Neck Department, Trieste, Italy

**Keywords:** Quality of life, Head and neck cancer, Surgery, Qualidade de vida, Câncer de cabeça e pescoço, Cirurgia

## Abstract

**Introduction:**

Treatments used in head and neck cancer greatly impact the physical, psychological and functional state of patients. Evaluation of quality of life has become an integral part of the treatment.

**Objective:**

This retrospective study evaluates features involved in changes in quality of life after major surgery for head and neck cancer within six months, according to self-reported outcomes.

**Methods:**

One hundred and thirty patients completed the University of Washington Quality of Life questionnaire one and six months after major surgery for head and neck cancer. A multivariate model was used to evaluate which diagnostic and therapeutic features were related to improvement of quality of life within a six-month period.

**Results:**

Significant improvement in most features related to quality of life was already recognizable at six months. Patients submitted to more invasive treatment had the biggest improvement in quality of life between time-points, as well as those patients with bigger tumors.

**Conclusion:**

After major surgery, patients may undergo fast recovery, with overall quality of life likely to improve in the short-term. Clinicians must be aware of the importance of dealing with treatment-related issues immediately after surgery, with hopeful possibility of on-the-upgrade results.

## Resumo

**Introdução:**

Os tratamentos adotados em câncer de cabeça e pescoço causam grande impacto nos estados físico, psicológico e funcional dos pacientes. A avaliação da qualidade de vida tornou-se parte integrante do tratamento.

**Objetivo:**

Este estudo retrospectivo avaliou os aspectos envolvidos nas mudanças da qualidade de vida pós-cirurgia de grande porte para câncer de cabeça e pescoço no período de seis meses, de acordo com os resultados autorrelatados.

**Método:**

Cento e trinta pacientes responderam ao Questionário de Avaliação de Qualidade de Vida da Universidade de Washington, um e seis meses após cirurgia de grande porte para câncer de cabeça e pescoço. Um modelo multivariado foi usado para avaliar quais características terapêuticas e diagnósticas estavam relacionadas à melhora da qualidade de vida no período de seis meses.

**Resultados:**

Melhora significativa na maioria dos aspectos relacionados à qualidade de vida já podia ser percebida em seis meses. Os pacientes submetidos a um tratamento mais invasivo apresentaram os melhores avanços na qualidade de vida entre os tempos de avaliação, bem como os pacientes portadores de tumores maiores.

**Conclusão:**

Após uma cirurgia de grande porte, os pacientes podem ter recuperação rápida, com melhora da qualidade de vida global em pouco tempo. Os médicos devem estar cientes da importância de lidar com questões relacionadas ao tratamento imediatamente após a cirurgia, devido à possibilidade de resultados melhores.

## Introduction

Oral/oropharyngeal squamous cell carcinoma (OSCC) is the fifth most frequent cancer among males and the seventh among females worldwide, with prevalence between 2% and 4% of all malignant tumors in Europe,^1^ and overall five-year survival rate of around 60%.^2^ The etiology of oral cancer is unknown, although various factors have been considered part of its development, such as smoking, alcohol and oncogenic viruses.^3^, ^4^ Being asymptomatic for a long time, OSCCs are often diagnosed at an advanced stage. Copious extirpation achieving tumor-free margins, as well as effective removal of affected/suspect lymph nodes, are vital steps to ensure long-term survival. Although treatment and reconstructive abilities have improved to such an extent that many patients can be fully integrated into society after treatment, OSCC can have a profound impact on the quality of life (QoL) of patients since its resection remains associated with disfigurement and dysfunctions.^5^, ^6^ For this reason, QoL is actually considered a multi-dimensional concept, which comprises the individual's perception of their state of health within the cultural context and value system where they live and in relation to their goals, socio-demographic parameters and social relations,^7^ and an integral part of the outcome of treatment.^8^

Many studies report that OSCC patients suffer from a worse cosmetic outcome, restricted independence in daily activities and recreation, serious deficits in chewing, swallowing and speech, and frequent mood and anxiety disorders.^9^ This is due to the fact that advanced OSCC requires highly destructive surgery with wide resections that involve transmandibular approaches, at times with resection, neck dissection and flap reconstruction as well as adjuvant radiotherapy (RT), with inevitable functional impairment.^10^

In this study we selected cancer patients and evaluated the improvement of QoL, expressed as “QoL outcomes”, through the University of Washington Quality of Life questionnaire (UW-QoL), in relation to diagnostic (age, sex, tumor site, tumor stage, nodes stage) and therapeutic (kind of cancer treatment, surgical approach and reconstructive technique) features after major surgery for OSCC. Time-points chosen were one month (T1) and six months (T6). Differently from the original version of UW-QoL, we propose a more focused analysis of the questionnaire in order to analyze in depth every single outcome investigated. To our knowledge, a few studies have considered variations of QoL parameters in the short term.^11^, ^12^

## Methods

The study was conducted at the Head and Neck Department of “Ospedale di Cattinara”, in collaboration with the Department of Oral Medicine and Pathology of “Ospedale Maggiore” (34100, Trieste, Italy), to evaluate the post-operative QoL of patients submitted to invasive oncological treatment for OSCC. A sample of 140 patients was selected.

Inclusion criteria were: surgical treatment between 2001 and 2008; with/without adjuvant RT, diagnosis of OSCC (according to TNM); ASA I, II, III.

Specifically, all the patients included in the present study had not started RT at T1 and had already finished RT at T6.

Exclusion criteria were: neo-adjuvant RT; ASA IV^12^; concomitant/previous chemotherapy.

Diagnostic and therapeutic features were recorded via examination of hospital records.

Evaluation of QoL was carried out using the UW-QoL questionnaire, which is composed of 16 multiple-choice questions. Each question has three to five multiple choices, as indicated in Table 1: the higher the score, the worse the related condition. The last three questions, regarding overall QoL, were considered as a whole. Questionnaire was handed out to patients at T1 and T6 after surgery.

**Table 1:** Outcomes of the study with the corresponding scores.

Outcome	Scores
QoL	0, very good; 1, good; 2, fair; 3, poor; 4, very poor
Pain	0, No pain; 1, mild no drugs; 2, severe but drug-responding; 3, severe and not drug-responding
Appearance	0, no change; 1, mild change; 2, bothering, not debilitating; 3, disfigured, limited in activity; 4, socially limited
Activity	0, no change; 1, sometimes limited; 2, tired, slowed down; 3, cannot go out; 4, must stay in bed
Recreation	0, no change; 1, still enjoy life; 2, cannot go out often; 3, limited in activities; 4, cannot do anything enjoyable
Swallowing	0, no change; 1, sometimes limited; 2, only liquid food; 3, cannot swallow
Chewing	0, no change; 1, only soft food; 2, only liquid food
Speech	0, no change; 1, can be understood over phone; 2, understood only by family and friends; 3, cannot be understood
Shoulder	0, no change; 1, stiff shoulder, activity not affected; 2, pan/weakness/work change; 3, cannot work
Taste	0, no change; 1, taste most foods; 2, limited; 3, cannot taste foods
Saliva	0, no change; 1, less saliva; 2, insufficient saliva; 3, no saliva
Mood	0, excellent; 1, good; 2, fair; 3, a bit depressed; 4, extremely depressed
Anxiety	0, not anxious; 1, little anxious; 2, anxious; 3, very anxious

Differently from the original version of the questionnaire,^13^ each question was considered a “QoL's outcome” and used to evaluate improvement between T1 and T6 through a regression model. This kind of analysis gives more focused results and isolates every single issue presented in the questionnaire, thus providing an immediate clinical implication. Being all patients Italians, the original version of the questionnaire was translated into Italian by a bilingual (Italian and English) translator.

The study was conducted after approval of the ethical committee (prot. No. 383/2009; 54/2009).

### Statistical analysis

The SPSS^®^ software 11.0 (Chicago, Illinois, USA) was employed. For each of the QoL outcomes, the significance of the difference between the time-points was evaluated through a Wilcoxon paired sign rank test.

Subsequently, the adjusted correlations of each of the diagnostic and therapeutic features with each of the QoL outcomes were evaluated by backward multiple logistic regressions, building 13 separate regression models. In particular, the changes in each of the QoL outcomes, computed as the difference between the scores recorded at T6 and T1 i.e. positive values for improved quality, were considered as the dependent variables. The explanatory variables (categories) entered in each model were: age, sex, tumor site; T stage; N stage; treatment type; neck dissection; surgical approach; reconstruction. The cut-off levels of significance were 0.05 and 0.10 for entry and removal, respectively. A *p*-value < 0.05 was considered statistically significant.

## Results

A total of 140 patients were included, and 130 (33.8% females and 66.2% males, with cumulative mean age of 65 ± 11) completed the questionnaire at both time points. Death of patient, incomplete questionnaire or missing a time point justified a drop out. Descriptive frequencies and univariate analyses are summarized in Table 2. Descriptive analysis of diagnostic and therapeutic features is presented in Table 2.

**Table 2:** Prevalence for each of the independent variables as count (%) (*n* = 130).

Parameter	Count (%)
*Gender*	
Male	57 (66.2)
Female	23 (33.8)

*Age*	
Male	65.9 ± 11.9
Female	65.8 ± 12.2

*T stage*	
T1	48 (36.9)
T2	43 (33.1)
T3	25 (19.2)
T4	14 (10.8)

*N stage*	
N0	80 (61.5)
N1	26 (20.0)
N2	22 (16.9)
N3	2 (1.5)

*Tumor site*	
Oral cavity	96 (73.8)
Oropharynx	34 (26.2)

*Treatment type*	
Surgical	71 (54.6)
Surgical and adjuvant RT	59 (45.4)
Demolitive transmandibular	30 (23.1)

*Surgical approach*	
Transoral	77 (59.2)
Conservative transmandibular	23 (17.7)
Demolitive transmandibular	30 (23.1)

*Reconstruction*	
Direct closure	70 (53.8)
Local flap	13 (10.0)
Pedicled flap	11 (8.5)
Microvascular free flap	36 (27.7)

*Neck dissection*	
No	38 (29.2)
Yes	92 (70.8)

Table 3 shows improvement of QoL's outcomes between time points (*p* < 0.001).

**Table 3:** Descriptive statistics for each of the QoL outcomes according to the time points (*n* = 130).

Outcome	1 month	6 months	Improved (count [%])
Overall QoL	3.0 (2.0–4.0)	2.0 (1.0–3.0)^a^	83 (63.8)
Pain	0.0 (0.0–0.5)	0.0 (0.0–0.0)^a^	29 (22.3)
Appearance	1.0 (0.0–2.0)	0.0 (0.0–1.0)^a^	62 (47.7)
Activity	0.0 (0.0–2.0)	0.0 (0.0–1.0)^a^	53 (40.8)
Recreation	0.0 (0.0–2.0)	0.0 (0.0–1.0)^a^	45 (34.6)
Swallowing	1.0 (1.0–2.0)	1.0 (0.0–1.0)^a^	73 (56.2)
Chewing	1.0 (0.0–2.0)	1.0 (0.0–1.0)^a^	53 (40.8)
Speech	1.0 (1.0–2.0)	1.0 (0.0–1.0)^a^	66 (50.8)
Shoulder	1.0 (0.0–2.0)	0.0 (0.0–1.0)^a^	51 (39.2)
Taste	0.0 (0.0–1.0)	0.0 (0.0–1.0)^a^	32 (24.6)
Saliva	0.5 (0.0–2.0)	0.0 (0.0–2.0)^a^	28 (21.5)
Mood	1.0 (0.75–2.0)	0.0 (0.0–1.0)^a^	65 (50.0)
Anxiety	1.0 (0.0–2.0)	0.0 (0.0–1.0)^a^	50 (38.5)

Diff., significance of the difference between the time points.

Level of significance:

a *p* < 0.001.

Multiple regression was performed to correlate QoL's improvement and diagnostic and therapeutic features for each outcome (Table 4).

**Table 4:** Multiple backward logistic regression models.

Parameter	Improved *n* (%)	OR (95% CI)
**Model 1: Outcome: QoL change**		
*Dissection*		
No	**27** (71.1)	**1**
Yes	**56** (60.9)	**0.3** (0.1–0.9)^a^
*Reconstruction*		
Direct closure	**44** (62.9)	**1**
Local flap	**5** (38.5)	**0.3** (0.9–1.0)^a^
Pedicled flap	**6** (54.5)	**0.9** (0.1–0.9)
Microvascular free flap	**28** (77.8)	**2.9** (1.1–7.8)^a^

**Model 2: Outcome: pain change**		
*Sex*		
Female	**12** (27.3)	**1**
Male	**17** (19.8)	**0.3** (0.1–1.0)^a^
*Site*		
Oral cavity	**17** (17.7)	**1**
Oropharynx	**12** (35.3)	**3.1** (1.2–8.6)^a^
*Treatment type*		
Surgery	**7** (9.9)	**1**
Surgery + RT	**22** (37.3)	**6.4** (2.3–17.4)^c^

**Model 3: Outcome: appearance change**		
*Surgical approach*		
Transoral	**23** (29.9)	**1**
Conservative transmandibular	**16** (69.6)	**3.4** (1.2–9.9)^b^
Demolitive transmandibular	**23** (76.7)	**5.5** (2.0–15.2)^a^
*Dissection*		
No	**7** (18.4)	**1**
Yes	**55** (59.8)	**4.0** (1.5–10.7)^a^

**Model 4: Outcome: activity change**		
*Sex*		
Female	**21** (47.7)	**1**
Male	**32** (37.2)	**0.3** (0.1–0.8)^a^
*Treatment type*		
Surgery	**18** (25.4)	**1**
Surgery + RT	**35** (59.3)	**3.4** (1.4–8.6)^b^
*Tumor stage*		
T1	**9** (18.8)	**1**
T2	**23** (53.5)	**2.9** (0.9–9.3)
T3	**17** (68.0)	**4.0** (1.1–14.5)^a^
T4	**4** (28.6)	**0.4** (0.1–2.0)
*Site*		
Oral cavity	**36** (37.5)	**1**
Oropharynx	**17** (50.0)	**2.4** (0.9–6.4)
*Surgical approach*		
Transoral	**22** (28.6)	**1**
Conservative transmandibular	**12** (52.2)	**1.9** (0.5–6.4)
Demolitive transmandibular	**19** (63.3)	**3.9** (1.2–13.3)^a^

**Model 5: Outcome: recreation change**		
*T stage*		
T1	**5** (10.4)	**1**
T2	**20** (46.5)	**5.1** (1.6–16.6)^a^
T3	**16** (64.0)	**7.0** (1.6–30.0)^a^
T4	**4** (28.6)	**1.2** (0.2–7.0)
*Reconstruction*		
Direct closure	**16** (22.9)	**1**
Local flap	**1** (7.7)	**0.2** (0.0–2.2)
Pedicled flap	**3** (27.3)	**0.7** (0.2–3.4)
Microvascular free flap	**25** (69.4)	**4.9** (1.6–15.1)^a^

**Model 6: Outcome: swallowing change**		
*Sex*		
Female	**30** (68.2)	**1**
Male	**43** (50.0)	**0.4** (0.2–0.8)^a^
*Dissection*		
No	**14** (36.8)	**1**
Yes	**59** (64.1)	**3.9** (1.7–9.0)^b^

**Model 7: Outcome: chewing change**		
*Reconstruction*		
Direct closure	**21** (30.0)	**1**
Local flap	**2** (15.4)	**0.4** (0.1–2.1)
Pedicled flap	**5** (45.5)	**1.9** (0.5–7.1)
Microvascular free flap	**25** (69.4)	**5.3** (2.2–12.7)^c^

**Model 8: Outcome: speech change**		
*Age*	–	**1.0** (0.9–1.0)
*Site*		
Oral cavity	**52** (54.2)	**1**
Oropharynx	**14** (41.2)	**0.5** (0.2–1.1)
*Reconstruction*		
Direct closure	**37** (52.9)	**1**
Local flap	**1** (7.7)	**0.1** (0.0–0.6)^a^
Pedicled flap	**5** (45.5)	**0.6** (0.2–2.3)
Microvascular free flap	**23** (63.9)	**1.6** (0.7–3.7)

**Model 9: Outcome: shoulder change**		
*Site*		
Oral cavity	**41** (42.7)	**1**
Oropharynx	**10** (29.4)	**0.4** (0.1–1.0)^a^
*Surgical approach*		
Transoral	**21** (27.3)	**1**
Conservative transmandibular	**15** (65.2)	**4.0** (1.3–12.0)
Demolitive transmandibular	**15** (50.0)	**1.7** (0.6–4.2)^a^
*Dissection*		
Yes	**5** (13.2)	**1**
No	**46** (50.0)	**5.2** (1.8–15.6)^b^

**Model 10: Outcome: taste change**		
*Age*	–	**1.0** (0.9–1.0)

**Model 11: Outcome: saliva**		
*Treatment type*		
Surgery	**9** (12.7)	**1**
Surgery + RT	**19** (32.2)	**3.2** (1.2–1.5)^a^
*Surgical approach*		
Transoral	**13** (16.9)	**1**
Conservative transmandibular	**10** (43.5)	**2.5** (0.8–7.4)
Demolitive transmandibular	**5** (16.7)	**0.6** (0.2–2.0)

**Model 12: Outcome: mood change**		
*Sex*		
Female	**26** (59.1)	**1**
Male	**39** (45.3)	**0.4** (0.2–1.0)^a^
*T stage*		
T1	**19** (39.6)	**1**
T2	**27** (62.8)	**3.3** (1.3–8.1)^b^
T3	**12** (48.0)	**1.5** (0.6–4.1)
T4	**7** (50.0)	**1.9** (0.6–6.6)

**Model 13: Outcome: anxiety change**		
*Sex*		
Female	**21** (47.7)	**1**
Male	**29** (33.7)	**0.3** (0.1–0.7)^b^
*Surgical approach*		
Transoral	**22** (28.6)	**1**
Conservative transmandibular	**16** (69.6)	**0.7** (0.3–1.7)^b^
Demolitive transmandibular	**12** (40.0)	**4.2** (1.3–13.9)
*Dissection*		
No	**8** (21.1)	**1**
Yes	**42** (45.7)	**0.4** (0.1–1.0)

A separate model was built for each outcome of QoL.

In each section defined as “model *n*”, diagnostic and therapeutic features are presented following the results of the multiple backward logistic regression, which isolates the relevant features among all the features considered. Results are presented both as number (and percentage) of improved cases, and through the odds ratio (OR).

Significance of each parameter is distributed as follows:

a *p* < 0.05.

b *p* < 0.01.

c *p* < 0.001.

Specifically, overall QoL improvement was less evident in patients who underwent neck dissection (OR 0.3) and more evident in patients who were submitted to invasive reconstructive techniques, such as microvascular free flap (OR 2.9). Local flap and pedicled free flap led to minor improvement (OR 0.3 and 0.9 respectively) instead. Concerning pain sensation, males improved less than females’ (OR 0.3) at T6. Patients who were submitted to adjuvant RT experienced a greater improvement of pain over time (OR 6.4). Moreover, a tumor in the oral cavity proved to be more painful at T6 than at T1 if compared to a tumor of the oropharynx (OR 3.1). Considering appearance, performance of neck dissection and type of surgical treatment determined an improvement between time points: the more invasive the intervention, the greater improvement experienced (*p* < 0.05). To mention, conservative and demolitive transmandibular interventions triggered greater amelioration of appearance when compared to transoral approaches (OR 3.4 and 5.5, respectively). Concerning the capacity of continuing daily activity, at T6 males felt more limited compared to females (OR 0.3), as did patients who underwent surgical intervention without adjuvant RT. In addition, patients diagnosed with T3 cancer resumed their daily activities more easily than patients with T1 cancer (OR 4.0), as did patients who were submitted to more invasive intervention (OR 3.9). Regarding recreation outcome, T2 (OR 5.1) and T3 (OR 7.0) tumors had major improvement between time points; the same happened for patients who went through more invasive surgical approaches (OR 4.9 for microvascular free flap). The maintenance of good swallowing capacity over time was higher in females than in males (OR 0.4) as well as in patients who were not submitted to neck dissection (OR 3.9). Regarding chewing capacity, greater improvement was evidenced after microvascular free flap (OR 5.3). In regards to speech capacity, older patients improved more than younger ones, and patients affected by oral cavity tumors had a better speech function at T6 than patients affected by oropharynx tumors (OR 0.5). According to reconstructive technique, the worst speech function at T6 was registered in patients operated through local flaps (OR 0.1). Shoulder pain improved more at T6 in patients operated for oral tumors compared to patients operated for oropharynx tumors (OR 0.4), as well as in patients who did not undergo neck dissection (OR 5.2). Demolitive transmandibular approach was associated to higher shoulder pain improvement over time (OR 1.7). In addition, adjuvant RT was linked to salivation's improvement at T6 (OR 3.2). Regarding mood, females improved more than males (OR 0.4) at T6. T2 tumor stage appeared to be the most influencing category for the same outcome (OR 3.3). According to anxiety, females experienced greater improvement than men (OR 0.3) at T6. Eventually, conservative transmandibular approach was tolerated worse (OR 0.7) than demolitive interventions.

## Discussion

The aim of this study was to determine which diagnostic and therapeutic features are involved in the post-operative improvement of QoL after major surgery for OSCC. The evaluation was performed in a short term of six months. Pre-operative evaluation was not considered, since the objective of the present investigation was to outline if there were significant differences between time points in the short term follow-up. Nevertheless, a baseline (before treatment) indicator would be of great interest, in conjunction to a psychological examination, which warrants future studies.

The UW-QoL questionnaire was chosen as it may offer a platform to identify those with significant problems in any of the 13 domains assessed.^14^ The modified version of the questionnaire we propose offers the possibility of isolating every single outcome as a specific feature, so that treatment is potentially more and more focused. Nevertheless, the multivariate model joins all the outcomes, giving a comprehensive and accurate reproduction of patients’ state of health. Recently, QoL measures have been incorporated into routine clinical oncology to monitor and screen individuals for significant dysfunction/problems, which they would be expected to encounter, thereby triggering healthcare intervention.^15^

Shown below, each of the diagnostic and therapeutic features has been analyzed separately and correlated to relevant QoL outcome, derived from the multivariate analysis. This approach may help the clinician to establish a hypothesis on how QoL could vary between one and six months after surgery, simply considering the diagnostic and therapeutic features.

### Sex/age

Men showed less improvement than females and proved to be more anxious and moody at T6. This can be correlated to the condition of distress, which is frequent in OSCC patients and more likely to develop in men.^16^ In regards to age, prolonged medical therapies, surgical complications and reconstructions must be carefully evaluated in elderly patients, since toleration could be lower. Unlike our expectations, QoL outcomes were not affected by age, in contrast to previous studies.^17^

### Tumor/node stage

Although bigger tumors are usually associated with more invasive interventions,^18^ probably leading to a worse immediate post-operative general status, our results support the hypothesis that even large masses can be operated with hopeful possibility of recovery within six months, allowing reintegration into society and return to daily activity in a short period of time. This result can be judged relevant, also considering that only one third of the patients experienced T3 and T4 stages in the fully evaluated sample. People who have malignancies are plagued with a variety of limiting symptoms. OSCC survivors suffer from deficits and facial disfigurement and experience several psychological concerns, including fear of recurrence and uncertainty with adjusting life beyond cancer.^19^ This condition can be defined as “distress”. Generally, distress strongly correlates to pain scores and occurrence of physical symptoms, as well as to bad mood/anxiety.^20^ Mood positive changes were already evident in our patients at T6, almost in all stages, most significantly in T2 patients. This occurs proportionally to acceptance of oneself, decline of symptoms and going back to daily activity. Consequently, psychological support may be recommended immediately after surgery. Surprisingly, no correlation between nodes’ stage and QoL domains was evidenced in our regression model. Actually, surgical techniques for selective node dissection are more and more advanced and tumor site/size-specific; this allows the best outcome of treatment.^21^

### Tumor site

In the present study, oral and oropharyngeal tumors were considered. In case of oropharyngeal tumors, improvement at T6 was registered for pain and activity, whereas speech and shoulder reported minor improvement in respect to oral tumors. This can be correlated to the fact that oropharyngeal tumors are often diagnosed at an advanced stage, due to difficulties in objective examination as well as to scarcity of symptoms, inevitably leading to demolitive intervention and long-lasting problems such as speech dysfunction. Although long-term rehabilitation is usually performed, complete speech recovery is usually not achievable, and speech intelligibility remains the major objective of treatment.^22^

### Treatment type

Patients treated for OSCC undergo different therapies, including surgery and neo-adjuvant/adjuvant RT/chemotherapy (CT). Surgery for OSCC is demolitive and invasive, due to anatomic characteristics of maxillofacial district as well as to evolution pattern of OSCC. The systemic metastases of OSCC occur via ipsilateral/contralateral lymphatic channels, so that lymph nodes’ dissection is performed to ensure that risk of occult metastasis’ risk is minimized. Serious complications as esthetic appearance, changing in daily activity, social concerns and functional impairment may occur.^23^ Patients usually undergo adjuvant RT in case of extracapsular spread and/or lymph nodes involvement.^24^ RT, in addition to surgical treatment, related to increased pain sensation and difficulty in daily activity. After surgical resection for oral cavity OSCC, adjuvant RT may be recommended for patients at higher risk for locoregional recurrence.^25^ Newer protocols recommend the association of adjuvant RT and CT after surgery, although this increases the risk of early and late cancer therapy-related side effects.^26^ In fact, RT is undoubtedly associated with acute side effects, such as mucositis, xerostomia, taste loss, swallowing difficulties, caries and trisma, as well as with chronic complications. In OSCC patients, xerostomia represents a late side effect of major concern for RT-treated patients. We have registered amelioration between time points in patients submitted to RT in association with surgery. Although subjective xerostomia seems to improve at T6 in RT-treated patients, only 32% of patients actually improved. This result is not reliable on the long term, since chronic complications usually start six to 12 months after RT. The present study did not considered the development of side effects related to RT, despite the fact that this could be of great interest for further investigation. In any case, some RT-related factors, such as pain sensation and performing of daily activity, are likely to ameliorate within six months, so clinicians should be concerned with patients’ needs according to the time period considered.

### Surgical approach

Several types of surgical approaches exist to manage OSCC. Actual techniques are “defect/patient orientated”; consequently, well defined as well as more and more surgically advanced reconstructive techniques have been developed to compensate surgical/functional defects.^27^ The complete conservation of mandibular bone continuity is achieved through “transoral approaches”. In case of pharyngeal tumors, it is not recommended, since it is associated with many post-operative complications such as hemorrhage, fistulae, dehiscence and nerve damage. On the contrary, the transmandibular approaches, although more demolitive, allow greater intraoperatory visibility and surgical management. Lots of advantages are associated with direct visualization of tumor, derived from a wide field of access such as good control of bleeding and light insertion; also, some studies evidenced less post-operative complications in pharyngeal tumors.^28^

In our study, patients who were submitted to demolitive surgery improved more in the short term than patients submitted to conservative intervention. This implies that although transmandibular intervention is far more invasive and surgically complicated, it is associated with tolerable recovery at T6. In addition, transmandibular interventions were associated with decreased shoulder pain at T6. Shoulder pain is frequently recognizable in patients who go through neck dissection due to tumor spread, but it is generally managed with physiotherapy and frequently fast recovering.^29^ After the diagnosis of cancer and in the perioperative phases, a sensation of distress may arise, and it is sometimes difficult to eradicate. The distress is related to fear of death, sensation of decline, pessimism.^30^ The improvement in various QoL outcomes, which we have presented so far, may contribute to general diminishing of anxious behavior.

### Reconstructive techniques

The head and neck region is one of the most difficult areas to reconstruct because it has complex anatomic, functional and physiologic interactions.^31^ Over the past decade, the use of free flap transfers in OSCC has led to remarkable advances in the reliability and the ultimate results of oromandibular reconstruction. Moreover, with the development of microsurgical free tissue reconstruction, it is commonly agreed that such patients can be rehabilitated earlier, thereby better readapting to their social environment. In agreement with many reports on the repair of head and neck defects by free flaps, 77.8% of our patients reported an improved overall QoL at T6 (*p* < 0.05). Functional impairment may be noticed among OSCC patients immediately after surgical intervention. In general, chewing and speech function improved between T1 and T6, but these factors were undoubtedly associated with reconstructive technique, since patients submitted to more complicated and invasive interventions experienced a greater improvement at T6. In agreement with literature, a six-month evaluation is reliable to assert that functional improvement can be expected after complex reconstructive techniques, with long-term stability.^32^ Reconstructive techniques that exploit free flap reconstructions have increased local control and long-term survival in cancer patients.^33^ Moreover, microvascular free tissue transfer has revolutionized the approach to the reconstruction of complex defects, providing a safe, reliable procedure to restore functionality and QoL for patients. Our results evidenced that a six-month period after surgery is a reliable indicator of QoL improvement. Many studies assert that reconstructive techniques are associated with worst QoL outcomes immediately after surgery, since such procedures imply pain, swelling and functional impairment.^34^, ^35^ At T6, microvascular free flap proved optimal for gradual improvement, at times with surpassing of pre-operative scores.^36^

### Neck dissection

While radical neck dissection was formerly seen as an essential measure for securing local tumor control and improvement of prognosis, it is nowadays replaced by selective neck dissection. This is associated with a comparably low morbidity and acceptable functional results, without having a negative impact on the prognosis.^37^ In any case, the dissection of the accessory nerve and great auricular nerve can easily cause long-term complications, including numbness, shoulder pain and motor dysfunction.^38^ In our study, patients who underwent neck dissection had a less evident QoL amelioration between T1 and T6, probably due to the abovementioned complications. Conversely, patients had gone through neck dissection experienced major improvement in appearance at T6 compared to patients not submitted to any dissection. Neck dissection is usually performed with an invasive approach, which leaves a visible and non-esthetic scar, but thanks to advanced surgical techniques, esthetic result becomes acceptable once tissue healing has occurred.^39^ Significantly, a corresponding improvement in anxiety sensation was registered at T6 in the same group of patients. Swallowing improved at T6 in patients who underwent neck dissection. This is in accordance with literature, which states that in case of neck dissection, swallowing function rehabilitation allows a continuous progress between T1 and T6.^40^

Of course, further investigations such as considering the development of acute/chronic side effects during cancer therapy and evaluation of long-term QoL and survival, would be complementary to the presented results, as would be inclusion of more explanatory variables such as evaluation of education, marital status, comorbidities, risk factors for OSCC. In our future plans, we have in mind to perform the same QoL analysis in a pre-operative phase, but also to add a psychological analysis through a dedicated questionnaire before and after treatment. We believe this would be of great help to perform the best supportive care possible, together with a prolonged follow up.

## Conclusions

This study discusses the issue of subjective post-operative QoL improvement after major surgery for OSCC within six months. In fact, despite the fact that the lack of evaluation of QoL before surgery could be considered a limitation, the addressed objective was the dynamic changing of patients’ referred QoL outcomes in the short term. Moreover, the modified version of the questionnaire we propose is more and more accurate in reproducing patients’ state of health between time points. Our results support the need for clinicians to establish adequate supportive care immediately after the end of surgical phases, accompanied by a patient-oriented and outcome-oriented therapy on the long term. The more invasive the intervention, the more likely it is that the situation will improve within a short period of time. In other words, patients affected by bigger tumors and submitted to more invasive interventions, especially with complicated reconstructions, usually experience severe QoL alterations immediately after major surgery, but are likely to improve quickly within the first six months.

## Conflicts of interest

The authors declare no conflicts of interest.
